# Hsa_circRNA_002144 promotes growth and metastasis of colorectal cancer through regulating miR-615-5p/LARP1/mTOR pathway

**DOI:** 10.1093/carcin/bgaa140

**Published:** 2020-12-21

**Authors:** Mengqiong Wu, Cancan Kong, Manni Cai, Weiwei Huang, Yiming Chen, Baochun Wang, Xin Liu

**Affiliations:** 1 Department of Gynecology Xiuying District, Haikou City, Hainan Province, China; 2 Department of Endoscopy Center Xiuying District, Haikou City, Hainan Province, China; 3 Department of Gastroenterology Xiuying District, Haikou City, Hainan Province, China; 4 Department of General Surgery, Hainan General Hospital, Hainan Affiliated Hospital of Hainan Medical University, Xiuying District, Haikou City, Hainan Province, China

## Abstract

CircRNAs (circular RNAs), recently identified as a critical regulator in tumorigenesis, participate in CRC (colorectal cancer) growth. However, the role of hsa_circRNA_002144 in CRC was poorly understood. Firstly, hsa_circRNA_002144 showed significantly elevation in both of CRC tissues and cell lines, and suggested closely associated with poor prognosis in patients. Secondly, data from functional assays revealed that silence of hsa_circRNA_002144 inhibited CRC progression with reduced cell viability, proliferation, migration and invasion, while enhanced cell apoptosis. In addition, *in vivo* CRC growth and metastasis were also suppressed by knockdown of hsa_circRNA_002144. However, CRC progression was promoted with over-expression of hsa_circRNA_002144. Thirdly, hsa_circRNA_002144 colocalized with miR-615-5p in the cytoplasm of CRC cells, and decreased miR-615-5p expression. Moreover, miR-615-5p could target LARP1 (La ribonucleoprotein 1, translational regulator). Lastly, the suppressive effects of hsa_circRNA_002144 knockdown on CRC progression were reversed by LARP1 over-expression. In conclusion, hsa_circRNA_002144 could sponge miR-615-5p to promote CRC progression through the regulation of LARP1, providing a therapeutic target for cancer intervention.

## Introduction

Colorectal cancer (CRC) is one of the most common cancers in the world (1). Although, the recovery rate is high after early CRC resection through multidisciplinary therapy (radiotherapy and systemic chemotherapy), high recurrence rate and distant metastasis still threaten a large proportion of patients (2). Efforts have been made to elucidate the genetic and molecular characteristics of CRC in order to predict prognosis and response to targeted therapy (3,4), however, tumor heterogeneity suggests the need for new CRC markers and therapeutic targets (5).

CircRNAs, highly conserved and stable covalently closed RNA transcript, formed by the reverse splicing of a single pre-mRNA with gene regulatory potential (6). Cumulative evidences suggest that circRNAs are closely associated with human diseases, especially cancer, and may be a better biomarker due to its abundance in tumor tissues or plasma and stability (7,8). Recently, circRNAs are involved in metabolic dysregulation and tumor development of CRC (9). For example, hsa_circ_001988 was decreased in CRC and closely associated with the clinical characteristics of patients (10). CircHIPK3 (11) or hsa_circ_0000069 (12) could promote CRC growth and metastasis, while circRNA CBL.11 suppressed CRC proliferation (13). Therefore, circRNAs could function as potential biomarkers and therapeutic targets for CRC. In addition, researches on more circRNAs associated with CRC progression may contribute to clinical value.

Hsa_circRNA_002144, a new circRNA produced by RPPH1 gene, was localized at chr14:20811282-20811431- and firstly found to be upregulated in hepatocellular carcinoma (14). Data from CircBase showed that hsa_circRNA_002144 was also upregulated in CRC plasma (15) and tissues (16). However, the detailed role of hsa_circRNA_002144 on CRC progression remains enigmatic. Generally, circRNAs could function as microRNA sponges to sponge miRNAs via the miRNAs response elements, and miRNAs could also target mRNAs to form a network responsible for CRC progression (17). Hsa_circRNA_002144 could sponge miR-326-mediated ETS transcription factors to promote cervical cancer progression (18). Hsa-miR-615-5p was reported to be a binding target of hsa_circRNA_002144 in hepatocellular carcinoma (14). MiR-615-5p was reported to suppress angiogenesis to regulate tumor microenvironment of CRC (19), and highly miR-615-3p expression was associated with poorly differentiated CRC (20). However, whether miR-615-5p was involved in hsa_circRNA_002144-mediated CRC progression, and the target mRNAs of miR-615-5p in CRC, remains elusive.

This study firstly verified the oncogenic role of hsa_circRNA_002144 in CCRC progression, the potential miR-615-5p-mediated mRNA target was then validated and suggested that hsa_circRNA_002144 functioned as a microRNA sponge to enhance CRC progression, providing the potential therapeutic target.

## Materials and methods

### Tumor tissues collection

Protocols were approved by the Ethics Committee of Hainan Affiliated Hospital of Hainan Medical University and in accordance with those of the 1964 Helsinki Declaration and its later amendments for ethical research involving human subjects. Sixty pairs of CRC and adjacent non-tumorous tissues were obtained from patients at Hainan Affiliated Hospital of Hainan Medical University. Patients with written informed consents were assigned with stage I, II, III and IV based on TNM (tumor-node-metastasis) classification.

### Cell culture

CRC cell lines (T84, LoVo, SNU-175, CL-34) and FHC (fetal human cells), purchased from the Chinese Academy of Sciences (Shanghai, China), were inoculated in RPMI 1640 medium (Transgene, Beijing, China) supplemented with 10% fetal bovine serum (Gibco, Carlsbad, CA, USA) at 37°C humidified incubator with 5% CO_2_.

### Cell transfection

Full length of hsa_circRNA_002144 and LARP1 were constructed into pcDNA3.1 vector (Invitrogen, Carlsbad, CA, USA). Mimics or inhibitor of miR-615-5p, as well as the negative controls (NC mimic, NC inhibitor) were synthesized by GenePharma (Suzhou, China). T84 or LoVo cells were transfected with pcDNA vectors, mimics or inhibitors via Lipofectamine 3000 (Invitrogen).

To establish stable T84 cells for knockdown of hsa_circRNA_002144, shRNAs targeting hsa_circRNA_002144 (sh 1# or 2#-circRNA) or the negative control (shNC) were constructed into pLKO.1 (Biosettia, San Diego, CA, USA). HEK-293T cells were then transfected with pLKO.1 shRNAs plasmid (1 μg), psPAX2 (750 ng) and pMD2.G (250 ng). Two days later, the specific lentiviruses (multiplicity of infection, 100) were harvested, and infected T84 cells under 8 mg/mL polybrene treatment. Stable T84 cells for knockdown of hsa_circRNA_002144 were obtained under 5 μg/mL puromycin treatment for 7 days.

### Cell viability

T84 or LoVo cells with different treatment (5 × 10^3^ cells/well) were seeded and incubated at indicated time (0, 24, 48, 72, 96 h). Additional 20 μL CCK8 solution (Dojindo, Tokyo, Japan) was added to the culture medium 2 h before the determination of absorbance at 450 nm by Epoch microplate Reader (BioTek, Winooski, VT, USA).

### Cell proliferation

T84 or LoVo cells with different treatment (5 × 10^2^ cells/well) were seeded and cultured with RPMI 1640 medium for 2 weeks. Cells were fixed in 10% formaldehyde and then stained with 0.1% crystal violet. Cell colonies were visualized under microscope (Olympus, Tokyo, Japan).

### Flow cytometer

T84 or LoVo cells with different treatment (1 × 10^6^ cells) were harvested, and resuspended with 100 μL binding buffer (KeyGEN BioTech, Jiangning, Nanjing, China) with ribonuclease (1 U/mL) and PI (propidium iodide, 0.5 µg) for 30 min. Cells were then incubated with 5 μL fluorescein isothiocyanate-conjugated annexin V, and then analyzed by FACS flow cytometer (Attune, Life Technologies, Darmstadt, Germany).

### Wound healing

T84 or LoVo cells with different treatment (5 × 10^3^ cells/well) were seeded for 24 h and scratched via a plastic pipette tip. Washed with phosphate-buffered saline buffer, cells were cultured for another 24 h and calculated the wound width under microscope (Olympus).

### Transwell assay

T84 or LoVo cells with different treatment (5 × 10^4^ cells) were incubated on upper chambers (BD Biosciences, San Jose, CA, USA) with serum-free medium coated with Matrigel. Medium with 10% fetal bovine serum was added to the lower chambers. Filters were removed 8 h later, and the invasive cells to the lower chambers were fixed in 100% methanol 24 h later. After staining with 0.1% crystal violet, cells were counted under microscope (Olympus).

### Fluorescence *in situ* hybridization

Fixed and permeabilized T84 or LoVo cells were hybridized with 8 ng/µL Cy3-labeled hsa_circRNA_002144 or Digoxigenin-labeled miR-615-5p probes (Invitrogen) overnight at 55°C under Fluorescent *In Situ* Hybridization Kit (RiboBio, Guangzhou, China). After incubation with specified secondary antibodies, cells were counterstained with 4′, 6-diamidino-2-phenylindole, and photographed by fluorescent microscope (Olympus inverted microscope IX71).

### Dual luciferase reporter assay

Wild-type or mutant sequences of hsa_circRNA_002144 or 3′-UTR of LARP1 were subcloned into pmirGLO luciferase reporter vector (GenePharma, Suzhou, China). T84 or LoVo cells were co-transfected miR-615-5p mimics or NC mimic with the vectors. Luciferase activities were performed 48 h after transfection.

### RNase R digestion

Total RNAs from T84 or LoVo cells were isolated via Trizol (Invitrogen). RNAs (5 μg) were incubated with RNase R (3 U/ μg; Epicentre Technologies, Madison, WI, USA) at 37°C for 15 min. The products were then identified via qRT-PCR.

### qRT-PCR

RNAs in cytoplasm and nucleus of T84 or LoVo cells were separated via PARIS Kit (Thermo Fisher, Waltham, MA, USA). miRNAs were extracted via miRcute miRNA isolation kit (Tiangen, Beijing, China). The isolated RNAs were reverse-transcribed into cDNAs, and qRT-PCR was conducted with SYBR Green Master (Roche, Mannheim, Germany). The condition was shown below: 95°C 20 s and 40 cycles of 95°C 10 s, 60°C 20 s, 70°C 5 s. GAPDH or U6 were used as endogenous controls, and data were analyzed by 2−∆∆CT method. The primer sequences were showed as below: GAPDH F: 5′-ACCACAGTCCATGCCATCAC-3′, GAPDH R: 5′-TCCACCACCCTGTT GCTGTA-3′, hsa_circRNA_002144 F: 5′-GGTCAGACTGGGCAGGAGAT-3′, hsa_circRNA_002144 R: 5′-GAGTGACAGGACGCACTCAG-3′, miR-615-5p F: 5′-TCCGATTCTCCCTCTGGGTC-3′, miR-615-5p R: 5′-GTGCAGGGTCCGAGGT-3′, LARP1 F: 5′-GCAACCTAAAGACACTAC-3′, miR-615-5p R: 5′-GTGCAGGGT CCGAGGT-3′, U6 F: 5′-CTCGCTTCGGCAGCACATA-3′, U6 R: 5′-AACGATTCACGAATTTGCGT-3′.

### Western blot

Proteins extracted from T84 or LoVo cells (30 µg) via radioimmunoprecipitation lysis buffer (Beyotime, Ningbo, China) were separated by sodium dodecyl sulfate–polyacrylamide gel electrophoresis. Proteins were then electro-transferred onto nitrocellulose membrane, and blocked in 5% bovine serum albumin. Followed by incubation overnight with anti-caspase3 and anti-cleaved caspase3 (1:2000), anti-E-cadherin and anti-N-cadherin (1:2000), anti-LARP1 and anti-mTOR (1:2500) or anti-GAPDH (1:3000) antibodies (Abcam, Cambridge, MA, USA) at 4°C, the membrane was incubated with horseradish peroxidase labeled secondary antibody (Abcam). Finally, the signals were determined by enhanced chemiluminescence (KeyGen, Nanjin, China).

### Mouse xenograft assay

Animal study was approved by the Ethics Committee of Hainan Affiliated Hospital of Hainan Medical University and in accordance with the National Institutes of Health Laboratory Animal Care and Use Guidelines. Twelve 5-week-old female BALB/c nude mice (20–25 g) were randomly divided into two groups (*n* = 6). T84 cells (5 × 10^6^/0.1 mL PBS) with stable knockdown of hsa_circRNA_002144 or shNC were subcutaneously injected into right flank of nude mice. Ten days later, tumor volume was calculated every two days. Twenty days later, the mice were killed with 40 mg/kg sodium pentobarbital, the tumor tissues were isolated, weighed and photographed. For detection of metastasis, the T84 cells were injected into NOD/SCID mice by tail vein. After intraperitoneal injection of luciferin (4 mg/50 μL phosphate buffered saline; Promega) for 10 min, the metastases were photographed by IVIS Lumina II system (Caliper LifeSciences, Hopkinton, MA, USA). Lung tissues were also isolated, and fixed in 10% formalin and embedded in paraffin. The sectioned lung tissues were subjected to H & E staining, and the representative images were observed via microscope (Olympus).

### Immunohistochemistry

Formalin-fixed and paraffin-embedded tumor sections from the mice were incubated in 3% H_2_O_2_ and then blocked in 5% bovine serum albumin. The sections were incubated overnight with anti-LARP1, anti-Ki67, anti-E-cadherin, anti-N-cadherin or anti-mTOR antibodies (Abcam). After incubation with horseradish peroxidase labeled secondary antibody, the slides were examined under a light microscope (Olympus) with counterstaining with hematoxylin.

### Statistical analysis

Data were expressed as mean ± SEM, and processed by SPSS 19.0 software. The statistical analyses were determined by Student's *t*-test, one-way analysis of variance, Pearson's chi-squared test, non-parametric test and the survival curves were analyzed by Kaplan–Meier method and log-rank test. Value of *P* < 0.05 was considered to be statistically significant.

## Results

### Expression of hsa_circ_002144 in CRC

**Figure 1.**
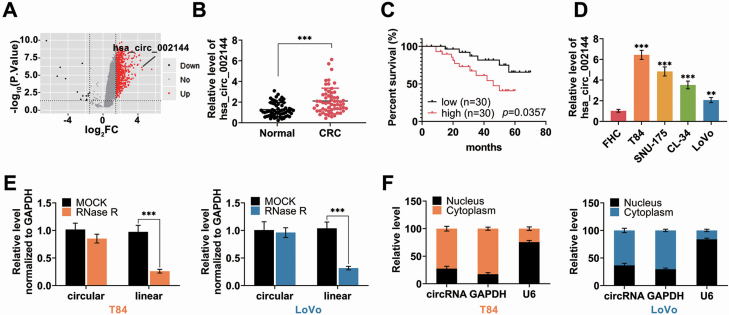
Expression of hsa_circ_002144 in CRC. (**A**) Volcano plot showed the differentially expressed hsa_circ_002144 between CRC and normal tissues from GSE126095 dataset. (**B**) The expression of circRNA hsa_circ_002144 in CRC tissues and adjacent non-cancer tissues detected by qRT-PCR (*n* = 60). (**C**) Overall survival analysis of CRC patients with high hsa_circ_002144 expression and low levels of hsa_circ_002144. (**D**) The expression of hsa_circ_002144 in CRC cell lines (T84, SNU-175, CL-34, Lovo) and FHC detected by qRT-PCR. (**E**) Hsa_circ_002144 was resistant to RNase R digestion compared with linear RPPH1 in both T84 and Lovo cells. (**F**) Expression of hsa_circ_002144, GAPDH and U6 in cytoplasm or nucleus of T84 and Lovo cells detected by qRT-PCR, suggesting the mainly cytoplasm subcellular localization of hsa_circ_002144. **P < 0.01, ***P < 0.001.

According to circRNA dataset of GEO database (GSE126095; 10 of patients and 10 of normal) (21), hsa_circ_002144 was significantly elevated in CRC patients (Figure 1A). qRT-PCR analysis confirmed the up-regulation of hsa_circ_002144 in 60 CRC tissues (Figure 1B). Group of highly hsa_circ_002144 expression (*n* = 30) was closely associated with shorter overall survival compared with group of lowly has_circ_002144 (*n* = 30) (*P* = 0.0357) through Kaplan–Meier curves analysis (Figure 1C), tumor size (*P* = 0.004), lymph node metastasis (*P* = 0.004), distant metastasis (*P* = 0.028) and TNM stage (*P* = 0.004) (Supplementary Table S1 is available at *Carcinogenesis* Online). Moreover, Univarite COX regression analysis demonstrated that hsa_circ_002144 was related to prognosis in patients with colorectal cancer (Supplementary Table S2 is available at *Carcinogenesis* Online). However, multivariate Cox regression analysis showed that hsa_circ_002144 was not an independent prognostic biomarker for colorectal cancer (Supplementary Table S2 is available at *Carcinogenesis* Online). These results suggested that hsa_circ_002144 might be involved in metastatic property of CRC. A significant up-regulation of hsa_circ_002144 was also verified in CRC cell lines (T84, LoVo, SNU-175, CL-34) (Figure 1D), and the circular nature of hsa_circ_002144 was validated in both of T84 and Lovo cells followed by RNase R digestion (Figure 1E). As shown in Figure 1F, hsa_circ_002144 was mainly expressed in cytoplasm of T84 and Lovo cells, suggesting that hsa_circ_002144 might function as miRNA sponger to regulate target genes in the nucleus of CRC cells.

### Hsa_circ_002144 promoted malignant behaviors of CRC

**Figure 2.**
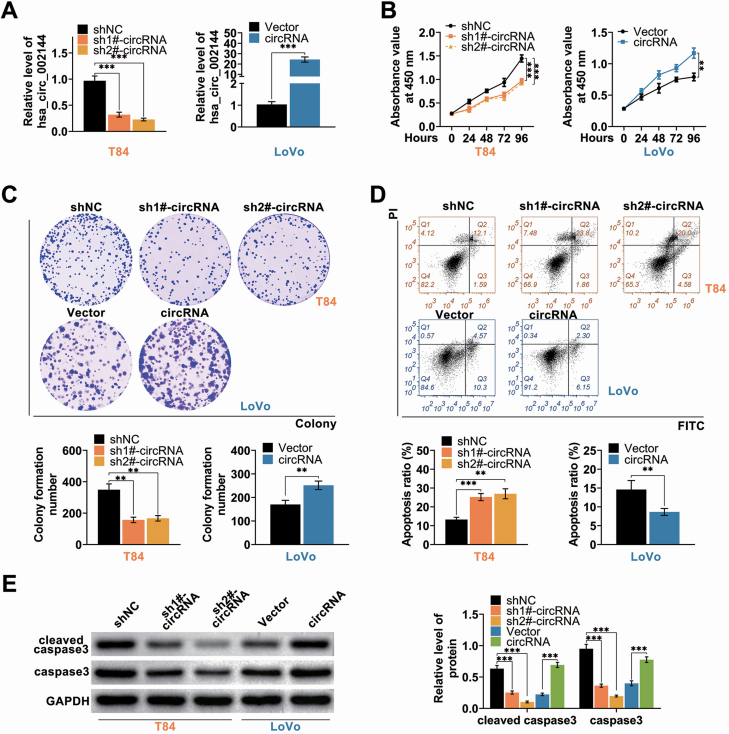
Hsa_circ_002144 promoted cell proliferation of CRC. (**A**) Transfection efficiency of pcDNA-hsa_circ_002144 in Lovo cells, and sh-hsa_circ_002144 1# or 2# in T84 cells, were detected by qRT-PCR. (**B**) The influence of hsa_circ_002144 on cell viability of T84 and Lovo cells detected by CCK8. (**C**) The influence of hsa_circ_002144 on cell proliferation of T84 and Lovo cells detected by colony formation assay. (**D**) The influence of hsa_circ_002144 on cell apoptosis of T84 and Lovo cells detected by flow cytometry. (**E**) The influence of hsa_circ_002144 on protein expression of caspase3 and cleaved caspase3 in T84 and Lovo cells detected by western blot. **P < 0.01, ***P < 0.001.

**Figure 3.**
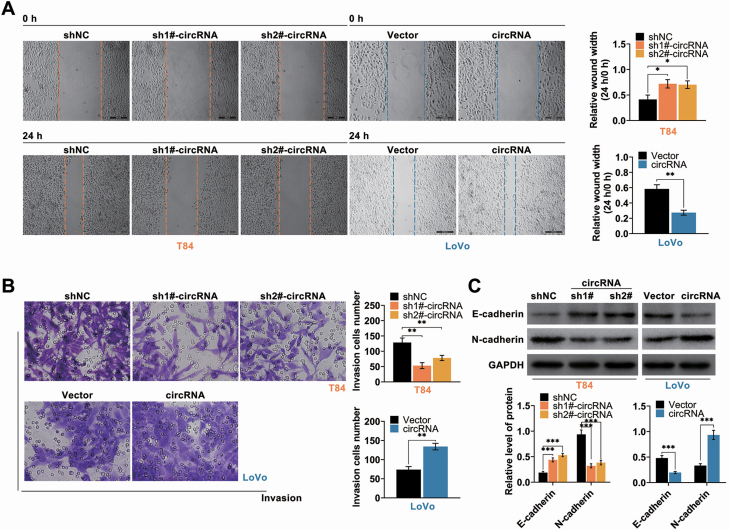
Hsa_circ_002144 promoted cell migration and invasion of CRC. (**A**) The influence of hsa_circ_002144 on cell migration of T84 and Lovo cells detected by wound healing assay. (**B**) The influence of hsa_circ_002144 on cell invasion of T84 and Lovo cells detected by transwell assay. (**C**) The influence of hsa_circ_002144 on protein expression of E-cadherin and N-cadherin in T84 and Lovo cells. *P < 0.05, **P < 0.01, ***P < 0.001.

Function roles of hsa_circ_002144 on CRC progression were then determined in T84 transfected with specific shRNAs targeting hsa_circ_002144 or Lovo transfected with pcDNA-hsa_circ_002144. The transfection efficiency was validated by qRT-PCR in Figure 2A. Over-expression of hsa_circ_002144 promoted cell viability (Figure 2B) and proliferation (Figure 2C) of Lovo cells, while knockdown of hsa_circ_002144 suppressed the cell viability (Figure 2B) and proliferation (Figure 2C). In addition, cell apoptosis was substantially decreased in Lovo cells (Figure 2D), while the apoptosis was significantly promoted in T84 cells (Figure 2D). Protein expression of caspase-3 and cleaved caspase-3 were decreased in T84 cells by knockdown of hsa_circ_002144, while increased in Lovo cells by over-expression of hsa_circ_002144 (Figure 2E). The metastatic capacities of T84 and Lovo were also suppressed by knockdown of hsa_circ_002144 (Figure 3A and B), while over-expression of hsa_circ_002144 promoted the metastatic capacities (Figure 3A and B)B. Protein expression of E-cadherin was increased in T84 cells with knockdown of hsa_circ_002144 (Figure 3C), and decreased in Lovo cells with over-expression of hsa_circ_002144 (Figure 3C). However, hsa_circ_002144 demonstrated a reversed effect on protein expression of N-cadherin compared with E-cadherin (Figure 3C). All these results indicated the suppressive effects of hsa_circ_002144 knockdown on malignant behaviors of CRC.

### Hsa_circ_002144 bind to miR-615-5p

**Figure 4.**
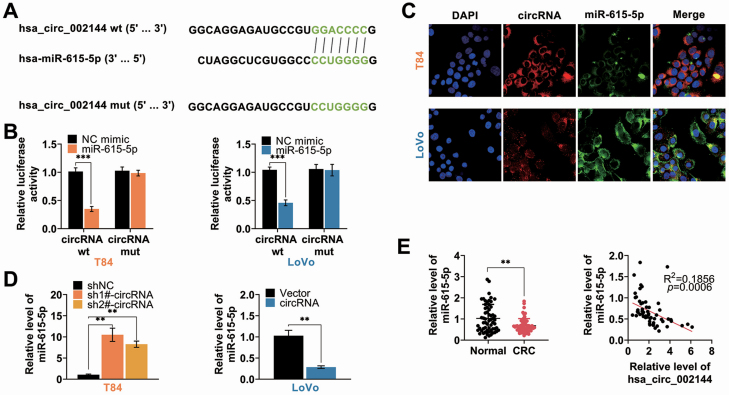
Hsa_circ_002144 bind to miR-615-5p. (**A**) The potential binding targets of hsa_circ_002144 predicted as miR-615-5p via circular RNA Interactome (https://circinteractome.nia.nih.gov/). (**B**) The influence of miR-615-5p mimics on luciferase activities of pmirGLO-wt-hsa_circ_002144 or pmirGLO-mut-hsa_circ_002144 in T84 and Lovo cells. (**C**) Subcellular localization of hsa_circ_002144 and miR-615-5p in T84 and Lovo cells via RNA-FISH. (**D**) The influence of hsa_circ_002144 on miR-615-5p expression in T84 and Lovo cells. (**E**) The expression of miR-615-5p in CRC tissues and adjacent non-cancer tissues detected by qRT-PCR (*n* = 60). Negative correlation between miR-615-5p and hsa_circ_002144 in CRC patients. **P < 0.01, ***P < 0.001.

Since hsa_circ_002144 was mainly expressed in cytoplasm of CRC cells, the sponging miRNA of hsa_circ_002144 was predicated as miR-615-5p (Figure 4A). Luciferase activity of pmirGLO hsa_circ_002144 wild-type luciferase reporter vector was substantially decreased in T84 and Lovo transfected with miR-615-5p mimics compared with NC mimic (Figure 4B). However, luciferase activity of pmirGLO hsa_circ_002144 mutant-type luciferase reporter vector did not show any obvious changes in T84 and Lovo transfected with either miR-615-5p mimics or NC mimic (Figure 4B). Fluorescence *in situ* hybridization indicated the colocalization between hsa_circ_002144 and miR-615-5p in cytoplasm of CRC cells (Figure 4C), furtherly confirming the direct binding ability between hsa_circ_002144 and miR-615-5p. Expression of miR-615-5p was reduced by hsa_circ_002144, while enhanced by hsa_circ_002144 knockdown (Figure 4D). In addition, down-regulation of miR-615-5p in CRC tissues (Figure 4E) revealed a significant negative correlation with hsa_circ_002144 in CRC patients (*P* = 0.0006) (Figure 4E). Therefore, hsa_circ_002144 binds to miR-615-5p in CRC cells.

### miR-615-5p inhibited LARP1

**Figure 5.**
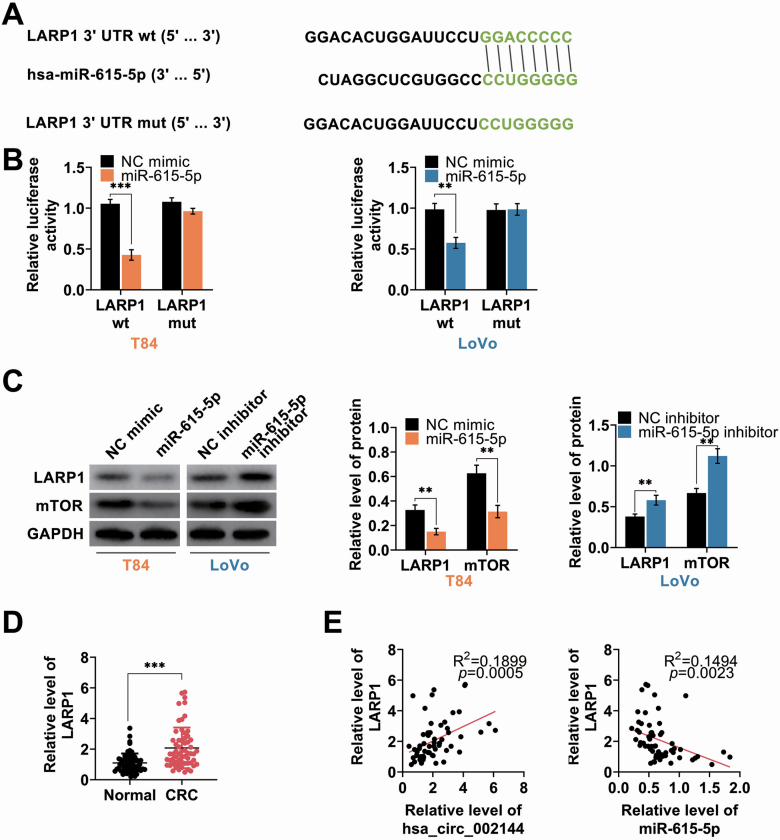
miR-615-5p inhibited LARP1. (**A**) The potential miR-615-5p binding targets predicted as LARP1 via Targetscan (http://www.targetscan.org/vert_71/). (**B**) The influence of miR-615-5p mimics on luciferase activities of pmirGLO-wt-LARP1 or pmirGLO-mut-LARP1 in T84 and Lovo cells. (**C**) The influence of miR-615-5p on protein expression of LARP1 and mTOR in T84 and Lovo cells detected by western blot. (**D**) The mRNA expression of LARP1 in CRC tissues and adjacent non-cancer tissues detected by qRT-PCR (*n* = 60). (**E**) Negative correlation between miR-615-5p and LARP1 in CRC patients. Positive correlation between hsa_circ_002144 and LARP1 in CRC patients. **P < 0.01, ***P < 0.001.

LARP1 was predicted as potential target of miR-615-5p (Figure 5A), and miR-615-5p mimics decreased the luciferase activity of LARP1 wild-type luciferase reporter vector (Figure 5B), while had no obvious effect on luciferase activity of LARP1 mutant-type luciferase reporter (Figure 5B). Moreover, proteins (Figure 5C) and mRNA (Supplementary Figure S2A is available at *Carcinogenesis* Online) expression of LARP1 and mTOR were reduced in T84 transfected with miR-615-5p mimics, and enhanced in Lovo transfected with miR-615-5p inhibitor (Figure 5C and Supplementary Figure S2A is available at *Carcinogenesis* Online). In addition, up-regulation of LARP1 in CRC tissues (Figure 5D) revealed a significant positive correlation with hsa_circ_002144 (*P* = 0.0005) (Figure 5E) and negative correlation with miR-615-5p (*P* = 0.0023) (Figure 5E) in CRC patients. Therefore, miR-615-5p binds to LARP1 in CRC cells.

### Hsa_circ_002144 promoted malignant behaviors of CRC through miR-615-5p-mediated LARP1

**Figure 6.**
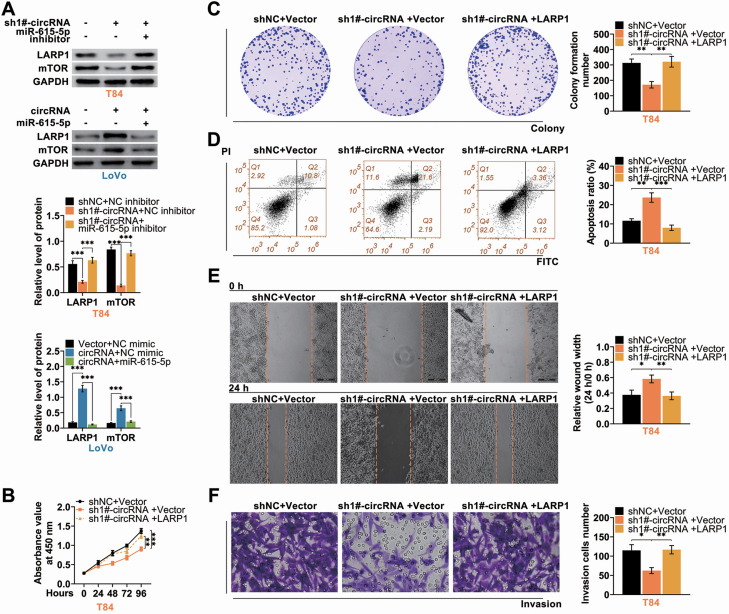
Hsa_circ_002144 promoted malignant behaviors of CRC through miR-615-5p-mediated LARP1. (**A**) The influence of hsa_circ_002144 and miR-615-5p on protein expression of LARP1 and mTOR in T84 and Lovo cells detected by western blot. (**B**) The influence of hsa_circ_002144 and LARP1 on cell viability of T84 cells detected by CCK8. (**C**) The influence of hsa_circ_002144 and LARP1 on cell proliferation of T84 cells detected by colony formation assay. (**D**) The influence of hsa_circ_002144 and LARP1 on cell apoptosis of T84 cells detected by flow cytometry. (**E**) The influence of hsa_circ_002144 and LARP1 on cell migration of T84 cells detected by wound healing assay. (**F**) The influence of hsa_circ_002144 and LARP1 on cell invasion of T84 cells detected by transwell assay. *P < 0.05, **P < 0.01, ***P < 0.001.

Rescue experiments were then conducted to investigate role of hsa_circ_002144/miR-615-5p/LARP1 on CRC progression. T84 was cotransfected with sh 1#-hsa_circ_002144 and miR-615-5p inhibitor, whereas Lovo was cotransfected with pcDNA-hsa_circ_002144 and miR-615-5p mimics. MiR-615-5p inhibitor could rescue the inhibitory effect of hsa_circ_002144 knockdown on protein (Figure 6A) and mRNA (Supplementary Figure S2B is available at *Carcinogenesis* Online) expression of LARP1 and mTOR, while miR-615-5p mimics rescued promotive effect of hsa_circ_002144 on protein expression of LARP1 and mTOR (Figure 6A and Supplementary Figure S2B is available at *Carcinogenesis* Online). Knockdown of hsa_circ_002144-suppressed cell viability (Figure 6B) and proliferation (Figure 6C) of T84 were reversed by over-expression of LARP1. However, over-expression of LARP1 attenuated hsa_circ_002144 silence-induced cell apoptosis (Figure 6D). In addition, suppressive effects of hsa_circ_002144 silence on cell migration (Figure 6E) and invasion (Figure 6F) were also reversed by over-expression of LARP1. Taken together, hsa_circ_002144 promoted malignant behaviors of CRC through miR-615-5p-mediated LARP1.

### Knockdown of hsa_circ_002144 suppressed CRC tumor growth

To further assess effect of hsa_circ_002144 on *in vivo* CRC tumor growth, xenograft mouse model was established and injected with T84 cells stably silence of hsa_circ_002144. Hsa_circ_002144 was significantly enhanced whereas miR-615-5p was reduced in sh 1#-hsa_circ_002144 group mice compared with shNC group (Supplementary Figure S1A is available at *Carcinogenesis* Online). Injection of T84 cells stably silence of hsa_circ_002144 suppressed tumor growth, as demonstrated by decrease of tumor volume and weight (Supplementary Figure S1B is available at *Carcinogenesis* Online). Moreover, tail vein injection of T84 cells stably silence of hsa_circ_002144 indicated suppressed CRC metastasis (Supplementary Figure S1C is available at *Carcinogenesis* Online), and the number of pulmonary metastatic nodules were also markedly decreased in mice injected with T84 cells stably silence of hsa_circ_002144 compared with shNC (Supplementary Figure S1D is available at *Carcinogenesis* Online). Immunohistochemical analysis showed decrease of LARP1, Ki-67, N-cadherin and mTOR, while increase of E-cadherin, in tissues from mice injected with T84 cells stably silence of hsa_circ_002144 compared with shNC (Supplementary Figure S1E is available at *Carcinogenesis* Online). These results showed that knockdown of hsa_circ_002144 suppressed CRC tumor growth and metastasis.

## Discussion

Increasing evidence has suggested that circRNAs could function as remarkable prognostic biomarkers of CRC (22). Moreover, the circular nature of circRNAs demonstrates resistance to RNAase, thus showing longer half-life than linear RNA and predicting as an attractive biomarker in CRC (23). Recently, circRNAs have also been verified to be associated with CRC development. ciRS-7-A not only functions as a promising prognostic biomarker in CRC, but also provides as a potential therapeutic target for CRC (24). Notably, a novel circRNA, hsa_circ_002144, was up-regulated in CRC (15,16), and hsa_circ_002144 could promote proliferation and invasion of cervical cancer, while suppressing apoptosis and autophagy (18). Nevertheless, the role and mechanism of hsa_circ_002144 on CRC growth required further exploration.

In line with previous reports (15,16), hsa_circ_002144 was dramatically up-regulated in CRC tissues, and this study for the first time revealed that highly hsa_circ_002144 expression was closely associated with metastatic properties of CRC, such as tumor size, lymph node metastasis, distant metastasis and TNM stage. Moreover, highly hsa_circ_002144 expression was also closely associated with shorter overall survival of CRC patients, suggesting that hsa_circ_002144 might serve as a potential biomarker for CRC prognosis.

Oncogenic role of hsa_circ_002144 in cervical cancer has been reported before (18), the present study showed that hsa_circ_002144 promoted cell viability, proliferation, migration and invasion of CRC cells, while induced the cell apoptosis. Moreover, the oncogenic role of hsa_circ_002144 in the invasion of CRC was associated with the promotion of epithelial–mesenchymal transition via an increase of N-cadherin and E-cadherin. Reports before have shown that CRC cells undergo epithelial-mesenchymal transition during local invasion (25,26). Reduce of E-cadherin and enhance of N-cadherin was shown to be associated with lymph node metastasis in CRC, and contribute to malignant progression of CRC (27). Results from *in vivo* tumor model revealed that silence of hsa_circ_002144 enhanced E-cadherin and reduced N-cadherin, thus inhibiting epithelial-mesenchymal transition of CRC. Moreover, knockdown of hsa_circ_002144 inhibited *in vivo* tumor growth and metastasis of CRC, suggesting potential clinical application of hsa_circ_002144 in CRC.

A potential circRNA–miRNA–mRNA network has been reported to be involved in CRC development (16). Hsa_circ_002144 has been shown to sponge miR-326 to regulate ETS transcription factor during cervical cancer progression (18). The underlying miRNA–mRNA network involved in hsa_circ_002144-mediated CRC progression was then clarified. Consistent with previous research that miR-615-5p was a potential binding target of hsa_circ_002144 (14), data from this study confirmed the direct interaction between hsa_circ_002144 and miR-615-5p in CRC. MiR-615-5p was widely considered as a tumor suppressor in hepatocellular carcinoma (28), ovarian cancer (29), non-small cell lung cancer (30), pancreatic ductal adenocarcinoma (31) and esophageal squamous cell carcinoma (32). Considering the angioregulatory role of miR-615-5p in CRC (19), miR-615-5p might also function as CRC tumor suppressor. Our results showed that miR-615-5p could target LARP1 to regulate CRC progression.

LARP1 is a conserved RNA-binding protein to control ribosome biogenesis (33), and participates in cancer cell survival (34), thus representing cancer therapeutic target (35). LARP1 has been reported to be prognosis biomarker of CRC, and silence of LARP1 inhibited cell proliferation of CRC (36). Here, this study showed that over-expression of LARP1 could counteract the suppressive effects of hsa_circ_002144 silence on CRC development. Therefore, hsa_circ_002144-miR-615-5p-LARP1 network was closely associated with CRC progression. Moreover, LARP1 could regulate mRNA stability and translation of mTOR to regulate cell growth and proliferation (37,38). LARP1/mTOR has been shown to contribute to epithelial cancer progression (39). Here, our results showed that knockdown of hsa_circ_002144 could promote miR-615-5p expression to decrease LARP1/mTOR during the suppression of CRC progression. mTOR is regulated by phosphatidylinositol 3-kinase/Akt pathway to participate in proliferation, angiogenesis and metastasis of CRC (40) and inhibition of mTOR could emerge as a potential strategy for cancer intervention of CRC (41–43). Whether phosphatidylinositol 3-kinase/Akt pathway was involved in hsa_circ_002144-miR-615-5p-LARP1-mTOR axis-mediated CRC progression should be investigated in further study.

In summary, knockdown of the oncogene, hsa_circ_002144, inhibited the development of HCC via sponging miR-615-5p-mediated LARP1-mTOR axis. This finding provided potential application of hsa_circ_002144 in CRC.

## Supplementary material

Supplementary data are available at *Carcinogenesis* online.

Figure S1. **Knockdown of hsa_circ_002144 suppressed tumor growth**. (A) Expression of hsa_circ_002144 or miR-615-5p in tissues from mice in sh1#-circRNA and shNC groups. (B) The effect of sh-hsa_circ_002144 on CRC tumor growth in xenograft tumor mice. The tumor volume and weight were calculated. (C) Represent bioluminescence images of mice in sh1#-circRNA and shNC groups. (D) H & E staining of lung tissues revealed morphologic features of lung tissues from mice in sh1#-circRNA and shNC groups. Tumor mass was indicated by arrows. (E) Immunohistochemistry staining was used to determine expression of LARP1, Ki-67, E-cadherin, N-cadherin and mTOR in tissues from mice in sh1#-circRNA and shNC groups. Scale bar: 200 μm. ** *P* < 0.01, *** *P* < 0.001.

Figure S2. (A) The influence of miR-615-5p on mRNA expression of LARP1 and mTOR in T84 and Lovo cells detected by qRT-PCR. (B) The influence of hsa_circ_002144 and miR-615-5p on mRNA expression of LARP1 and mTOR in T84 and Lovo cells detected by qRT-PCR.

Table S1. Relationship between hsa_circ_002144 and clinico-pathological parameters of CRC patients.

Table S2. COX analysis of hsa_circ_002144 in colorectal cancer.

bgaa140_suppl_Supplementary_Table_S1

bgaa140_suppl_Supplementary_Table_S2

bgaa140_suppl_Supplementary_Figure_S1

bgaa140_suppl_Supplementary_Figure_S2

## Data Availability

All data generated or analyzed during this study are included in this published article.

## Abbreviations

CircRNA: circular RNA
CRC: colorectal cancer
LARP1: La ribonucleoprotein 1
