# Supplementary material for: Hsa_circRNA_002144 promotes growth and metastasis of colorectal cancer through regulating miR-615-5p/LARP1/mTOR pathway
Source: Carcinogenesis. 2020 Dec 21;42(4):601–10. doi: 10.1093/carcin/bgaa140 (PMC8086769; doi:10.1093/carcin/bgaa140)
Supplement: bgaa140_suppl_Supplementary_Table_S1 [file bgaa140_suppl_supplementary_table_s1.docx]

Table S1 Relationship between hsa_circ_002144 and clinico-pathological parameters

| Parameters | Number of  patients | hsa_circ_002144 expression | | *P* value |
| --- | --- | --- | --- | --- |
|  |  | Low (< median) | High (≥ median) |  |
| Number | 60 | 30 | 30 |  |
| Gender |  |  |  |  |
| Male | 33 | 16 | 17 | 0.795 |
| Female | 27 | 14 | 13 |  |
| Age (years) |  |  |  |  |
| ≥Mean (65) | 32 | 14 | 18 | 0.301 |
| <Mean (65) | 28 | 16 | 12 |  |
| Tumor site |  |  |  |  |
| Colon | 38 | 20 | 18 | 0.592 |
| Rectum | 22 | 10 | 12 |  |
| Tumor size (cm) |  |  |  |  |
| ≥5 | 25 | 7 | 18 | 0.004** |
| <5 | 35 | 23 | 12 |  |
| Pathological T category | |  |  |  |
| T1-T2 | 26 | 14 | 12 | 0.602 |
| T3-T4 | 34 | 16 | 18 |  |
| Lymph node metastasis | |  |  |  |
| N0 | 33 | 22 | 11 | 0.004** |
| N1-2 | 27 | 8 | 19 |  |
| Distant metastasis |  |  |  |  |
| M0 | 47 | 27 | 20 | 0.028* |
| M1 | 13 | 3 | 10 |  |
| TNM stage |  |  |  |  |
| I | 16 | 13 | 3 | 0.011* |
| II | 15 | 8 | 7 |  |
| III | 16 | 6 | 10 |  |
| IV | 13 | 3 | 10 |  |
| Differentiation |  |  |  |  |
| Well | 10 | 8 | 2 | 0.071 |
| Moderate | 35 | 17 | 18 |  |
| Poor | 15 | 5 | 10 |  |
