# Supplementary material for: Hsa_circRNA_002144 promotes growth and metastasis of colorectal cancer through regulating miR-615-5p/LARP1/mTOR pathway
Source: Carcinogenesis. 2020 Dec 21;42(4):601–10. doi: 10.1093/carcin/bgaa140 (PMC8086769; doi:10.1093/carcin/bgaa140)
Supplement: bgaa140_suppl_Supplementary_Table_S2 [file bgaa140_suppl_supplementary_table_s2.docx]

Table S2 Cox model analysis of overall survival

| **Parameters** | ***P*-value** | **Relative risk (95% CI)** |
| --- | --- | --- |
| **Univariate** |  |  |
| Gender | 0.735 | 0.851(0.334±2.165) |
| Age (years) | 0.156 | 0.473(0.168±1.33) |
| Tumor site | 0.425 | 1.473(0.568±3.819) |
| Tumor size (cm) | 0.000 | 0.133(0.043±0.411) |
| Pathological T category | 0.794 | 0.883(0.347±2.247) |
| Lymph node metastasis | 0.028 | 2.926(1.123±7.622) |
| Distant metastasis | 0.094 | 2.881(0.835±9.942) |
| TNM stage | 0.031 | 1.692(1.048±2.733) |
| Differentiation | 0.310 | 1.473(0.698±3.108) |
| hsa_circ_002144 expression | 0.045 | 0.365(0.136±0.979) |
| **Multivariate** |  |  |
| Tumor size (cm) | 0.005 | 0.142(0.036±0.557) |
| Lymph node metastasis | 0.488 | 2.022(0.276±14.816) |
| TNM stage | 0.509 | 0.685(0.222±2.108) |
| hsa_circ_002144 expression | 0.458 | 0.669(0.231±1.935) |
