# Supplementary figures and images for: Hsa_circRNA_002144 promotes growth and metastasis of colorectal cancer through regulating miR-615-5p/LARP1/mTOR pathway
Source: Carcinogenesis. 2020 Dec 21;42(4):601–10. doi: 10.1093/carcin/bgaa140 (PMC8086769; doi:10.1093/carcin/bgaa140)

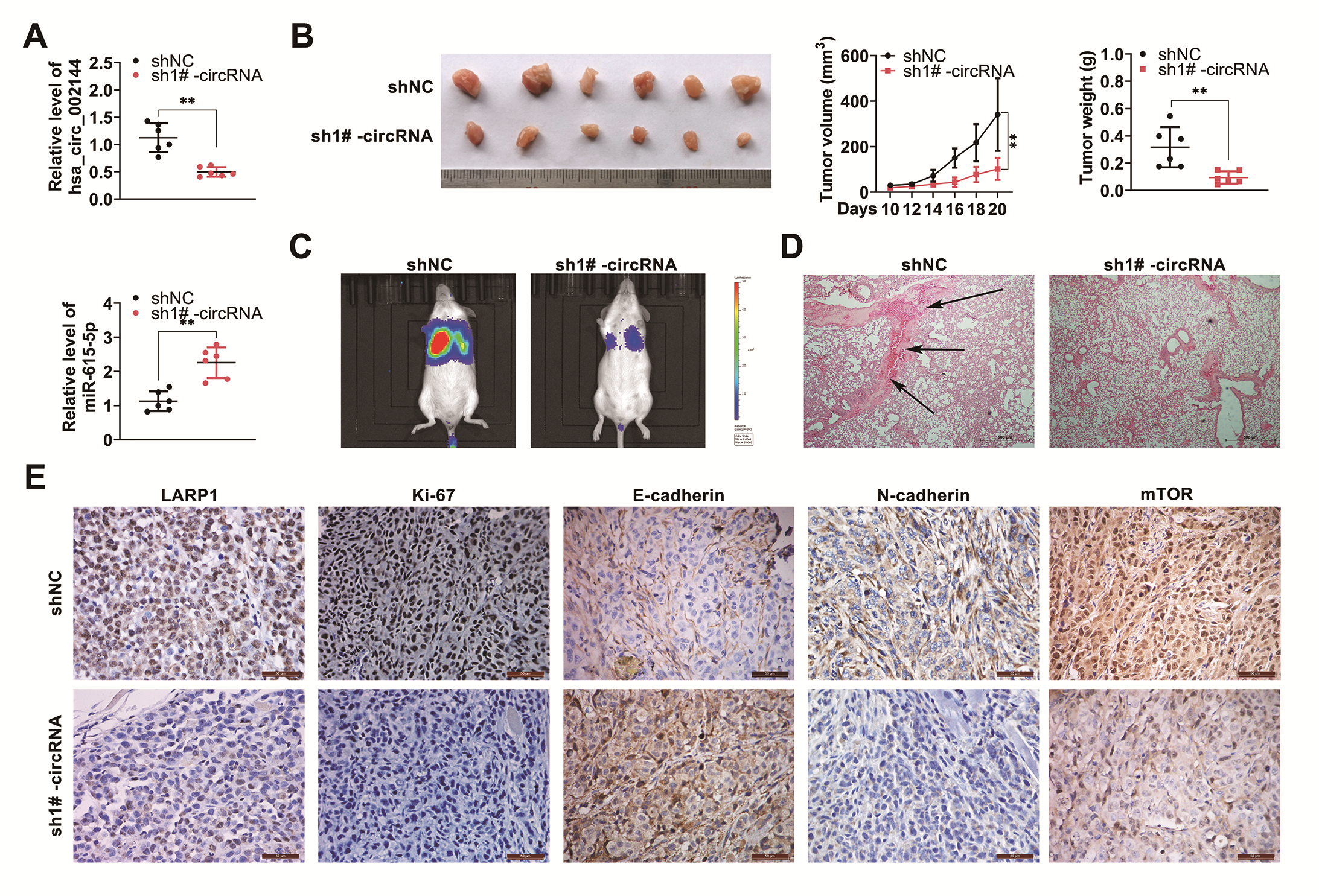

Supplement: bgaa140_suppl_Supplementary_Figure_S1 [file bgaa140_suppl_supplementary_figure_s1.png]

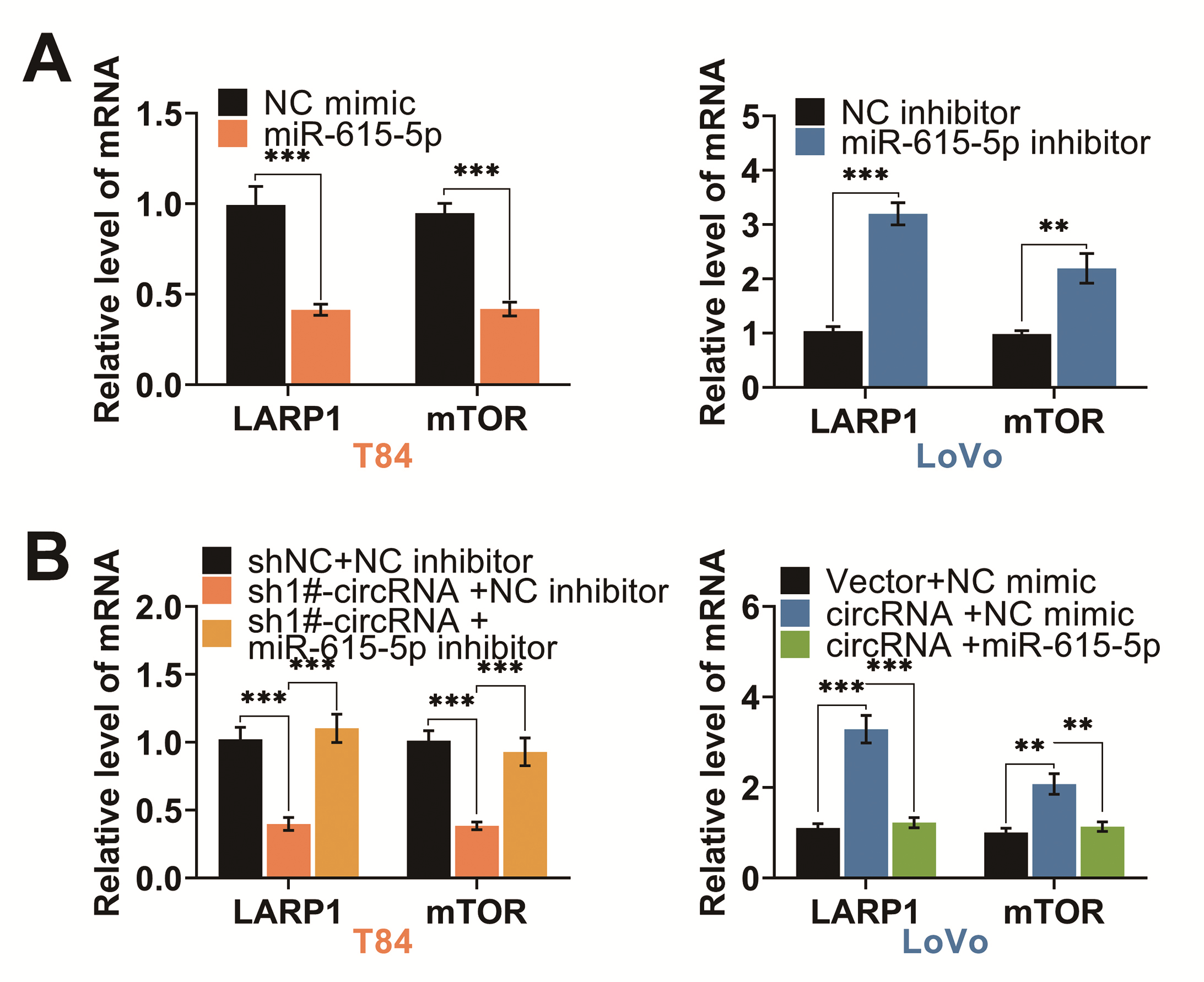

Supplement: bgaa140_suppl_Supplementary_Figure_S2 [file bgaa140_suppl_supplementary_figure_s2.png]
